# Donor-reactive T cells and innate immune cells promote pig-to-human decedent xenograft rejection

**DOI:** 10.21203/rs.3.rs-6474835/v1

**Published:** 2025-04-22

**Authors:** Farshid Fathi, Nathan Suek, Benjamin Vermette, Kevin Breen, Yasmeen S. Saad, Constanza Bay, Christopher A. Parks, Jeffrey Stern, Karen Khalil, Jacqueline Kim, Ian S. Jaffe, Imad Aljabban, Ekaterina Novikova, Elizabeth Severa, Ramin Sedaghat Herati, Lars Burdorf, Adam D. Griesemer2, Robert A. Montgomery, Megan Sykes

**Affiliations:** Department of Medicine, Columbia Center for Translational Immunology, Columbia University Medical Center, Columbia University; New York, NY, USA.; Department of Medicine, Columbia Center for Translational Immunology, Columbia University Medical Center, Columbia University; New York, NY, USA.; Department of Medicine, Columbia Center for Translational Immunology, Columbia University Medical Center, Columbia University; New York, NY, USA.; Department of Medicine, Columbia Center for Translational Immunology, Columbia University Medical Center, Columbia University; New York, NY, USA.; Department of Medicine, Columbia Center for Translational Immunology, Columbia University Medical Center, Columbia University; New York, NY, USA.; Department of Medicine, Columbia Center for Translational Immunology, Columbia University Medical Center, Columbia University; New York, NY, USA.; Department of Medicine, Columbia Center for Translational Immunology, Columbia University Medical Center, Columbia University; New York, NY, USA.; Department of Surgery, Transplant Institute, New York University Langone Health; New York, NY, USA.; Department of Surgery, Transplant Institute, New York University Langone Health; New York, NY, USA.; Department of Surgery, Transplant Institute, New York University Langone Health; New York, NY, USA.; Department of Surgery, Transplant Institute, New York University Langone Health; New York, NY, USA.; Department of Surgery, Transplant Institute, New York University Langone Health; New York, NY, USA.; Department of Medicine, NYU Grossman School of Medicine; New York, NY, USA.; Department of Medicine, NYU Grossman School of Medicine; New York, NY, USA.; Department of Medicine, NYU Grossman School of Medicine; New York, NY, USA.; United Therapeutics Corporation; Research Triangle Park, NC, USA; Department of Surgery, Transplant Institute, New York University Langone Health; New York, NY, USA.; Department of Surgery, Transplant Institute, New York University Langone Health; New York, NY, USA.; Department of Medicine, Columbia Center for Translational Immunology, Columbia University Medical Center, Columbia University; New York, NY, USA.

## Abstract

Xenotransplantation of pig organs is a promising solution to the organ shortage; however, rejection remains a major obstacle. Pig-to-human decedent transplantation provides an opportunity to study immune barriers to xenotransplantation experimentally. We tracked donor-reactive T cell dynamics in a 61-day pig-to-human decedent thymokidney xenotransplant. Xenogeneic donor-reactive T cell clones (XDRTCCs) identified using high-throughput *TCRB CDR3* sequencing expanded markedly in peripheral blood in association with apparent antibody-mediated rejection (AMR). Single-cell RNA and TCR sequencing of leukocytes from the xenograft showed XDRTCC infiltration and effector transcript expression during AMR. Additionally, γδ and NK cells with cytotoxic effector phenotypes were prominent in the rejecting xenograft. These data suggest that improved suppression of innate immunity and T cell responses might enhance the success of xenotransplantation.

Kidney transplantation is the only definitive treatment for end stage renal disease (ESRD), but the number of available organs falls far short of demand (1). Xenotransplantation, the transplantation of organs across species, offers a potential solution to this shortage. Pigs are a particularly attractive source animal candidate, given their suitable organ size, physiology, and modifiable genetics (2).

Both adaptive and innate immune responses pose barriers to xenotransplantation. In adaptive responses, T cells recognize xenoantigens through the direct pathway, in which recipient T cells recognize donor antigens on donor antigen-presenting cells (APCs), and through the indirect response, in which xenoantigens are processed and presented by recipient APCs. The indirect pathway has been reported to be more potent for xenoresponses than alloresponses (3, 4). The innate xenoresponse includes natural antibodies that can cause hyperacute or delayed antibody-mediated rejection when directed against pig carbohydrate antigens (2, 5–7). In addition, natural killer (NK) cells are prominent components of the cellular infiltrates in xenografts (8, 9), potentially because pigs do not express inhibitory ligands that prevent human NK cell activation (1).

Preclinical xenotransplantation studies have largely relied on non-human primates (NHPs). Although quite valuable, studies in these animals may not fully replicate the human xenoresponse. The pig-to-human decedent model has allowed investigation of these responses, primarily in short-term (up to 7 days) studies (10–12). Recently, we carried out a longer-term study in the pig-to-human decedent model that allowed unprecedented investigation of the physiological and immunological outcomes of pig kidney transplantation for 61 days in a human body (13).

Our prior studies have shown that in the xenotransplantation setting, pig thymus induces central tolerance to the source pig of recipient αβ T cells, resulting in xenograft tolerance (14–17). We now report on the immune response in a brain-dead human recipient transplanted with an GGTA1 (α-Gal) knockout (KO) pig (GalSafe^™^)composite thymokidney (UThymoKidney^™^) graft and followed for 61 days. We establish a method to first identify human anti-pig xenoreactive T cell clones, then track those clones in the decedent’s peripheral blood and kidney biopsies. We show that xenoreactive T cell clones (XDRTCCs) selectively expanded in the periphery following the transplant and were detected in a perinephric lymphocele as early as post-operative day (POD) 14, where they demonstrated an early activated transcriptional profile. XDRTCCs detected later in the kidney xenograft during an episode of acute antibody-mediated rejection (AMR) demonstrated mixed effector and exhausted transcriptional profiles. We also detected infiltrates of effector γδ T cells and NK cells with marked effector function in the kidney biopsy. To our knowledge, this is the first comprehensive analysis of the innate and adaptive cell-mediated rejection response in pig to human xenotransplantation. The results have important implications for immunosuppression design and highlight the need to better suppress T cell responses.

## Results

### Establishing direct and indirect Mixed Lymphocyte Reactions (MLR) to identify xenoreactive T cell clones

**Figure 1:**
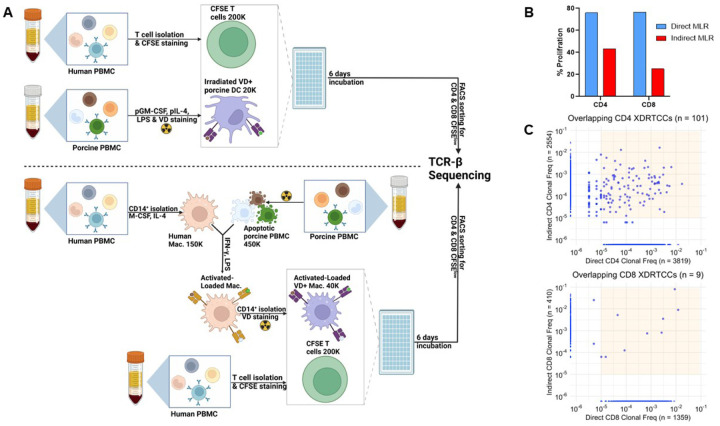
Characterization of healthy donor indirectly and directly xenoreactive T cell repertoire *in vitro*. (**A, upper panel**) Method for direct xenogeneic mixed lymphocyte reaction (MLR). Porcine DC are co-cultured with CFSE-labeled purified human T cells. After 6 days, proliferated T cells, identified as CFSE^low^, are sorted by flow cytometry, genomic DNA is extracted, and high throughput *TCRB* CD3 sequencing is carried out. (**A, lower panel**) Method for indirect xenogeneic MLR. Pig PBMCs are irradiated with 100 Gy to induce apoptosis and loaded into differentiated, sorted human macrophages. Loaded macrophages are MACS sorted for CD14 expression and combined with CFSE-labeled purified T cells. Proliferated CFSE^low^ T cells are sorted by flow cytometry, genomic DNA is extracted, and high throughput *TCRB* CDR3 sequencing is carried out. (**B**) Direct and Indirect MLRs induce proliferation of both CD4 and CD8 cells. (**C**) Clonal frequency plots of divided T cells following high-throughput TCR sequencing of the CD4 and CD8 response to indirect (y axis) and direct (x axis) stimulation by porcine antigens. Clonotypes that are unique to one pathway appear along the y or x axis (for indirect and direct respectively). Clonotypes found in both sequence sets are depicted within the yellow-shaded area.

To study the human anti-pig indirect and direct T cell response, we first established *in vitro* assays. For the direct response, peripheral blood mononuclear cells (PBMCs) of an *α*-Gal KO SLA-HH pig (18) were differentiated to dendritic cell (DCs). These porcine DCs were co-cultured for 6 days with CFSE-labelled sorted CD3^+^ human T cells purified (> 90% purity) from a healthy donor (Fig.1A, upper panel). We observed 75.9% and 76.4% proliferation of CD4 and CD8 T cells, respectively, at the end of the coculture period (Fig.1B). To set up the indirect response (Fig.1A, lower panel), we purified human T cells (>90% purity) and differentiated human macrophages from the same healthy volunteer. Macrophages were activated and loaded with pig antigens and were co-cultured with CFSE-labelled T cells. We observed 43.2% and 25.1% proliferation of CD4 and CD8 T cells, respectively (Fig.1B).

Semi-direct presentation of intact donor MHC-peptide complexes on recipient APCs is an additional pathway that has been described for alloresponses (3). If such complexes were picked up and presented intact by recipient macrophages in the indirect MLR described above, they might be expected to expand the same T cell clones as those activated by direct presentation on porcine donor APCs. Therefore, we performed bulk *TCRB* CDR3 sequencing on the T cell response from each pathway to determine the extent of clonal overlap. We observed that most clones appeared along the y (indirect) or x (direct) axis in the frequency correlation plots, indicating that these clones were unique to one or the other response (Fig.1C). In the CD4 response, only 101 overlapping clones were detected between 3,819 direct and 2,554 indirect clones (Fig.1C, upper panel). In the CD8 response, only 9 overlapping clones were detected between 1,359 direct and 410 indirect clones (Fig.1C, lower panel). Taken together, these data demonstrate that these indirect and direct assays identify largely distinct, non-overlapping xenoreactive T cell repertoires.

### TCRB CDR3 sequencing demonstrates expansion of T cell clones in circulation of human decedent recipient of porcine thymokidney transplant

**Figure 2:**
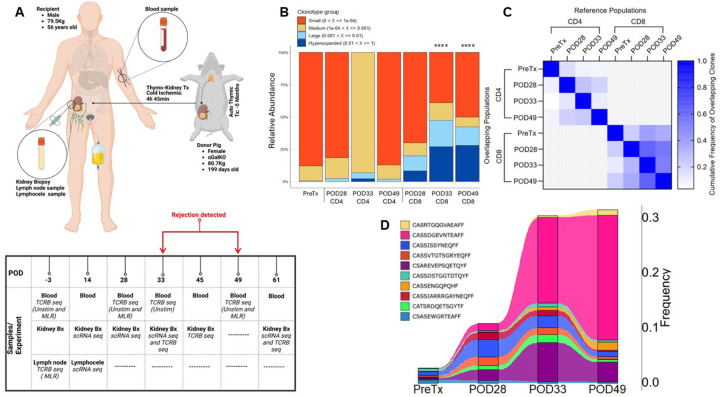
Overview of long-term thymokidney xenotransplant, sampling, and *TCRB* CDR3 sequencing of peripheral blood T cell DNA over time. (**A**) Schematic overview of the xenotransplantation experiment. Blood, kidney, and lymphocele samples were obtained from the decedent human and source pig pre- and post-transplantation. T cells were sorted from unstimulated (unstim) populations or from CFSE^low^ cells in MLRs. (**B**) Clonal expansion of circulating CD4 and CD8 T cells over time. CD4 and CD8 T cells were not separated in pre-transplant FACS sort. Fisher’s exact test was used to compare proportion of hyperexpanded clones compared to pre-Tx baseline, ****P < 0.0001 (**C**) Cumulative frequency of clones shared between timepoints. The reference population on the x-axis is the population whose cumulative frequency is plotted, and the overlap frequency is shown for the populations on the y-axis. The pre-transplant repertoire has been included by estimating the number of CD4 and CD8 templates using flow cytometric data (Extended Data Fig.10C). (**D**) Frequency of the top 5 CD8 clones from every sequenced post-operative timepoints. Each color represents the frequency of a specific clonotype at that timepoint. Pre-transplant frequencies were determined by normalizing the frequency of individual CD8 clones within the CD3^+^ pre-transplant population to the proportion of CD8^+^ cells within the CD3^+^ gate as determined by flow cytometry (Extended Data Fig. 10C). Some of the top 5 clonotypes are shared across the timepoints, resulting in fewer than 15 unique clones.

A porcine thymokidney graft was constructed by autologous thymus transplantation under the kidney capsule of an *α*-Gal KO source pig (United Therapeutics Corporation and its subsidiary, Revivicor, Inc.) (Fig.2A). Our prior studies have shown that pig thymus induces central tolerance to the source pig of human αβ T cells, resulting in xenograft tolerance (14–17, 19). After 5 months, the thymokidney was transplanted into a brain-dead, decedent recipient who had undergone bilateral native nephrectomy (13). After successful thymokidney transplantation, the decedent recipient underwent frequent peripheral blood and kidney biopsy sampling at various time points (Fig.2A). Throughout the study, the thymokidney demonstrated life-sustaining physiologic function, supporting normal hemodynamics and electrolyte balance, as described in detail elsewhere (13). Induction and maintenance immunosuppression are summarized in Table S1. Complement deposition gradually increased in the tissue, culminating in an episode of biopsy-confirmed AMR on POD33. This episode was treated with 5 alternate-day plasmaphereses along with the C3 inhibitor pegcetacoplan. Serum creatinine rose again at POD49 after concluding these treatments, and repeat biopsy showed ongoing AMR. Another cycle of 5 plamapheresis treatments and rATG was shown to have completely reversed the AMR on a repeat biopsy on POD56 (13). The creatinine returned to baseline and the xenograft remained free of rejection until termination of the study on POD61.

We monitored circulating T cell clones in the decedent recipient by performing high-throughput *TCRB* CDR3 sequencing on peripheral blood T cell DNA extracted pre- and post-transplant timepoints. The number of templates (i.e. total *TCRB* CDR3 sequences obtained) and unique clones identified in each sample are summarized in Extended Data Fig.1A. Over time, there was an increase in the abundance of hyperexpanded clonotypes, defined as clones with greater than 1% frequency. At POD33 and POD49, CD8 hyperexpanded clonotypes accounted for more than 25% of the CD8 repertoire, a statistically significant increase from the pre-transplant baseline (Fig.2B). Among CD8 T cells, the R20, the proportion of clonotypes representing 20% of templates (20), decreased, while clonality, whereby higher scores indicate less clonal diversity, increased (Extended Data Fig.1B-C). The cumulative frequency of overlapping clones confirmed that the same immunodominant CD8 clones were detected at each post-transplant timepoint (Fig.2C). The top 5 expanded clones made up only 3% of the pre-transplant CD8 repertoire compared to more than 30% by POD49 (Fig.2D). One clone in particular, shown in pink, comprised less than 0.1% of the pre-transplant repertoire and expanded to over 20% of the repertoire by POD49. Thus, marked expansion of a few immunodominant CD8 clones occurred over time post-transplant. Extended Data Fig.1B-C demonstrate less marked post-transplant loss of CD4 diversity.

### Selective post-transplant expansion of XDRTCCs

We identified directly and indirectly xenoreactive T cell clones *in vitro*, then tracked them in the long-term pig-to-human decedent thymokidney recipient. Using pre-transplant PBMCs from the decedent, cell clumping of PBMCs precluded the identification of large numbers of clones in unstimulated cells and direct and indirect MLRs against the source pig. To identify additional XDRTCCs, we also utilized a pre-transplant lymph node and post-operative PBMCs as responders, including a direct MLR using sorted POD28 PBMC CD3^+^ cells and another with POD49 recipient PBMCs. CFSE^low^ CD4^+^ and CD8^+^ T cells from each MLR were sorted for *TCRB* CDR3 sequencing. To exclude bystander proliferation effects and sorting errors, only clones with a frequency in the divided population at least twice that of their pre-transplant unstimulated frequency were considered XDRTCCs (Table S2). The indirect and direct pathways of xenostimulation expanded largely non-overlapping XDRTCCs (Extended Data Fig.2A-D). In total, we identified 1,058 CD4^+^ and 386 CD8^+^ XDRTCCs (Table S2).

**Figure 3:**
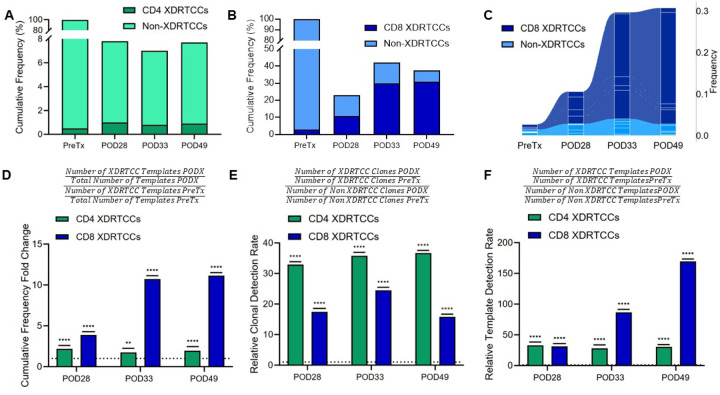
XDRTCCs demonstrate selective expansion and enrichment in the blood over time. (**A, B**) Cumulative frequency of CD4 (A) and CD8 (B) XDRTCCs and non-XDRTCCs at each timepoint. Pre-transplant frequencies were determined by normalizing the frequency of individual CD4 and CD8 clones within the CD3^+^ pre-transplant population to the proportion of CD4^+^ and CD8^+^ cells within the CD3^+^ gate, respectively, as determined by flow cytometry (Extended Data Fig. 10C). (**C**) Designation of the top 5 expanded CD8 clones (see Fig.2D) according to XDRTCC or non-XDRTCC status at each timepoint post-transplant. (**D**) Cumulative frequency fold change of both CD4 and CD8 post-transplant XDRTCCs as normalized to their pre-transplant baseline. The formula is shown. (**E**) Relative clonal detection rate of both CD4 and CD8 XDRTCCs compared to non-XDRTCCs identified pre-transplant. The formula is shown. (**F**) Relative template detection rate of both CD4 and CD8 XDRTCCs compared to non-XDRTCCs identified pre-transplant. The formula is shown. For Figures D, E and F, a dotted line is plotted at y = 1 to represent the cutoff between XDRTCC enrichment (>1) or depletion (<1). The statistics establish significance between the numerator and denominator in every case (post-transplant to pre-transplant XDRTCC cumulative frequency for Figure D, and XDRTCC to non-XDRTCC enrichment for Figures E and F). *P < 0.05, ** P < 0.01, ***P < 0.001, ****P < 0.0001, by Z test.

Post-transplant PBMC samples were compared for the frequency of XDRTCCs. CD4 XDRTCCs comprised less than 2% of templates in the post-transplant peripheral blood populations but were increased above pre-transplant baseline (Fig.3A). CD8 XDRTCCs demonstrated more striking expansions, constituting 10% of the total CD8 repertoire at POD28 and about 30% at POD33 and POD49, both of which were established rejection timepoints (Fig.3B). Individual expanded XDRTCCs could be traced across multiple time points, with one single clone comprising over 20% of the repertoire at POD49 (Fig.3C). This highly expanded XDRTCC is the pink clone from Fig.2D. All 5 top clones in Fig.2D were XDRTCCs. Normalizing the total quantity of circulating XDRTCCs post-transplant to their pre-transplant baseline revealed clear XDRTCC enrichment, with increases up to 11-fold at POD49 for CD8 cells (Fig.3D).

We assessed whether circulating XDRTCCs detected at each timepoint were directly or indirectly xenoreactive or were undefined because they were either expanded in both responses or only detected in the POD49 MLR. At all timepoints, a substantial proportion of detected CD4 XDRTCC clones were directly xenoreactive and a smaller proportion was indirectly reactive. The proportion of CD8 XDRTCC templates from the direct response increased from 15% pre-transplant to 73% by POD49 due to clonal expansions of directly reactive XDRTCCs (Extended Data Fig.3A-B), including the pink clone in Fig.2D.

Since clonal expansions may have been induced in part by lymphopenia produced by induction treatment with rATG prior to transplant, we compared the expansion of XDRTCCs to that of non-XDRTCCs, which were defined as clones present in the pre-transplant blood but not in any proliferated MLR population. Comparing the rates of post-transplant versus pre-transplant XDRTCCs and non-XDRTCC detection, XDRTCC-specific expansion is readily apparent at both clonal and template levels (Fig.3E–F). These relative expansions (>150-fold for CD8 XDRTCCs at POD 49) were most striking when calculated at the template level. The actual proportion of XDRTCC clones detected post-transplant was statistically significantly greater than that of non-XDRTCCs for both CD4s and CD8s, substantiating XDRTCC-specific expansion (Extended Data Fig.3C-D). Moreover, amongst TCRs in the post-transplant peripheral blood, a greater average number of CD8 XDRTCC unique nucleotide sequences converged to the same amino acid sequence than was observed pre-transplant, suggesting that specific antigens were driving expansion of donor-reactive clonotypes following the transplant (Extended Data Fig.3E). Of note, following the transplant, the average nucleotide to amino acid sequence ratio also increased significantly for non-XDRTCCs among CD4 and CD8 subsets, consistent with antigen-driven expansion of both sets of clones during post-transplant lymphopenia. In contrast, clones that were identifiable as neither XDRTCCs nor non-XDRTCCs post-transplant maintained a nucleotide to amino acid sequence ratio close to 1 (shown as “remainder” in Extended Data Fig.3E). In sum, our studies demonstrate marked and antigen-driven expansion of circulating XDRTCCs and, to a lesser extent, of non-XDRTCCs following pig-to-human decedent xenotransplantation.

For POD 33, 45, and 61, two 10 um-thick sections of formalin-fixed, paraffin-embedded xenograft biopsy specimens were cut and submitted for mRNA-based TCRb recovery (RepSeq+ assay, iRepertoire, Inc). The number of unique *TCRB* CDR3 RNA transcripts retrieved is summarized in Extended Data Fig.4A. XDRTCCs were detectable in all biopsies, including two on POD33, sixteen on POD45 and one on POD61. Among the nine unique CD8 XDRTCCs in the POD45 biopsy, five clones matched the immunodominant circulating CD8 XDRTCCs identified in Fig.2D and 3C. Their respective RNA frequencies in the biopsy are shown in Extended Data Fig.4B. Integration of all TCR data across all samples and time points identified five clones that could be traced from the POD14 lymphocele, then blood, then kidney biopsy during the AMR episode, and then finally study termination at POD61 (Extended Data Fig.4C). Of note, these five clones were the immunodominant circulating CD8 XDRTCCs shown in Fig.2D and 3C.

### Detection of XDRTCCs with effector function in the kidney biopsy during an antibody-mediated rejection episode

To further characterize T cells in the xenograft itself, we isolated leukocytes from kidney biopsies obtained at various time points (Fig.2A), MACS sorted them for human CD45^+^ cells, and carried out single-cell RNA and TCR sequencing. Additionally, cells were collected from a lymphocele (POD14L) that surrounded the transplanted pig kidney at POD14. The numbers of leukocytes submitted for single-cell RNA sequencing are summarized in Table S3. The number of pig and human transcripts in each cell were quantified. Most cells expressed almost entirely human or pig transcripts, making the species of origin easily distinguishable. All pig cells were removed from downstream analysis (Extended Data Fig.5A-C).

**Figure 4:**
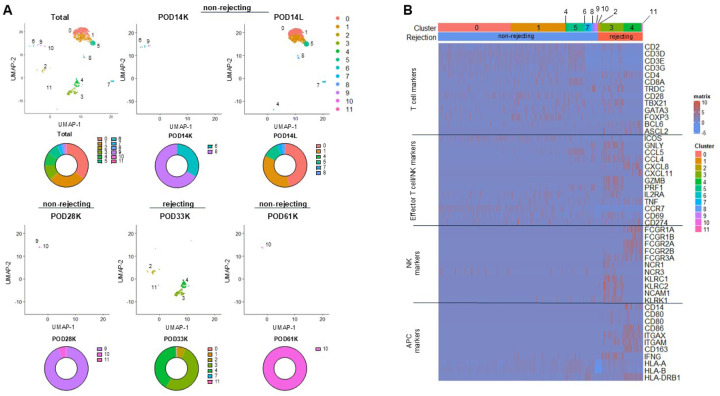
Single-cell RNA sequencing of xenograft leukocytes demonstrates upregulation of cytotoxic populations during rejection. (**A**) Integrated UMAP showing single-cell RNA sequencing results on hCD45^+^ sorted cells from POD14 kidney biopsy (POD14K), POD14 lymphocele (POD14L), POD28 kidney biopsy (POD28K), POD33 kidney biopsy (POD33K), and POD61 kidney biopsy (POD61K). Cluster numbering is color-coded. When split by POD, specific clusters predominate in specific samples. For example, Clusters 0, 1, 6 and 9 are primarily found in POD14L, whereas Clusters 2, 3, 4, 5, and 12 are predominantly found in the POD33 kidney specimen. (**B**) Expression of T cell, effector T and NK cell, NK cell, and APC-associated RNA species in each cluster and specimen in panel A.

Single-cell RNA sequencing data from all samples were processed and integrated by the standard Seurat pipeline and projected on a single Uniform Manifold Approximation and Projection plot (UMAP) (Fig.4A). To identify immune cells, we interrogated the single-cell RNA sequencing data for immune cell markers. The non-rejecting kidney biopsy specimens (POD14K, 28K, 61K) did not reveal gene expression patterns consistent with human T cells, NK cells, or APCs. In contrast, the POD14L and POD33 rejecting kidney specimen contained RNA species characteristic of T cells (e.g. CD3E, CD4, CD8A, CD28), NK cells (e.g. FCGR3A, NCR1, NCAM1), effector T cells and NK cells (e.g. GZMB, PRF1, TNF, GNLY) and APCs (e.g. CD80, CD86, HLA-DRB1) (Fig.4A–B, Extended Data Fig.5D-F).

Specific clusters identified on the UMAP segregated with specific specimens. For example, Clusters 0, 1, 5, and 8, containing mainly T cells, were predominantly detected in POD14L, whereas Clusters 2, 3, 4, and 11, containing T cells, NK cells and APCs, were predominantly detected in the POD33 kidney biopsy, during the episode of acute humoral rejection (Fig.4A-B, Extended Data Fig.5D-F). Cluster 3, which was uniquely present in the rejecting sample and included both T and NK cells), demonstrated high expression of cytotoxicity genes, including GZMB, PRF1, TNF, and GNLY (Fig.4B). Activated APC populations with upregulated costimulatory ligands, Fc receptors, chemokine receptors, HLA-DR, TNF and integrins were predominantly detected in Cluster 4 (Fig. 4B). Additionally, B cells (Cluster 7) were detected only in the POD14L (lymphocele) specimen (Extended Data Fig.5D-F).

**Figure 5:**
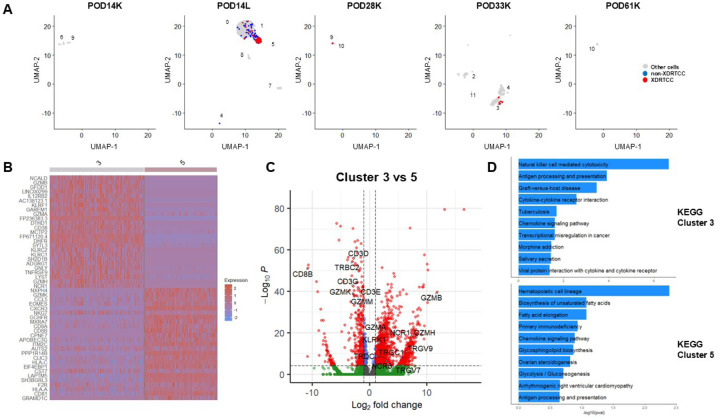
XDRTCCs demonstrate a cytotoxic phenotype, particularly during rejection at POD33. (**A**) Distribution of XDRTCCs (red) across POD14K, POD14L, POD28K, POD33K, and POD61K samples demonstrates that the majority are found in POD14L and POD33K samples. (**B, C**) Differential gene expression in clusters 3 and 5, containing the most XDRTCCs, demonstrates upregulation of effector cell-associated genes in both clusters 3 (GZMB, GZMH, FASLG) and 5 (GZMK, CCL5, EOMES, CXCR3). In addition, Cluster 3 demonstrates upregulation of γδ T cell genes TRGV9, TRGC, and NK cell-associated gene NCR3, whereas Cluster 5 demonstrates upregulation of adaptive T cell genes including CD8B and TRBC2. (**D**) KEGG pathway analysis of the top differentially expressed genes demonstrate NK cell-mediated cytotoxicity as the top pathway for Cluster 3.

We interrogated the single-cell TCR sequencing data for XDRTCCs. XDRTCCs were detected in POD14L and POD28 and POD33 kidney biopsies. The largest number of XDRTCCs was detected prior to rejection in POD14L in Cluster 5. Importantly, XDRTCCs in the rejecting specimen on POD33 were detected exclusively in Cluster 3 (Fig.5A, Extended Data Fig.6A-B), the major cluster containing cytotoxic T cells. The majority of XDRTCCs found in the lymphocele and kidney biopsy were directly xenoreactive CD8 T cells, although the lymphocele also included directly and indirectly xenoreactive CD4 cells and POD33K included directly donor-reactive CD4 clones and undefined CD8 clones (Extended Data Fig.6C-D). Of note, several clones within the POD33 kidney biopsy belonged to the most expanded clonotypes detected in the blood (Fig.2D).

XDRTCCs in Cluster 5 (at POD14L) differentially expressed several markers of activation and effector differentiation, including GZMK, EOMES, and CCL5 (Fig.5B, 5C). One XDRTCC in this cluster also expressed CXCR5, ICOS, and CD40L, consistent with a Tfh phenotype (Extended Data Fig.7A). Some XDRTCCs were later detected in Cluster 3 in the biopsy at POD33, during the episode of acute rejection (Extended Data Fig.7B), suggesting that initial T cell activation in lymphatic tissues preceded migration into the xenograft and rejection. We compared Cluster 3, containing the thirteen XDRTCCs upregulated in the biopsy specimen during rejection at POD33, to the main T cell-containing cluster, Cluster 5 in POD14L (Fig.5A). Cluster 3 differentially expressed cytotoxic genes GZMA, GZMB, GNLY, PRF1 and GZMH as well as IFNG, while Cluster 5 expressed GZMK (Fig.5B–C, Extended Data Fig.8A). CD8B and TRBC2 were prominent in Cluster 5, consistent with its αβ T cell content. In contrast, Cluster 3 also contained TRGC1, TRDC, and NCR1 (Fig.5B–C) in addition to the XDRTCC αβ TCRs mentioned above (Fig.5A), suggesting that Cluster 3 included γδ T cells and NK cells. Indeed, using pathway analysis, the top upregulated pathway in Cluster 3 corresponded to NK cell-mediated cytotoxicity, suggesting a contribution of innate immune cells to cytotoxicity (Fig.5D).

**Figure 6:**
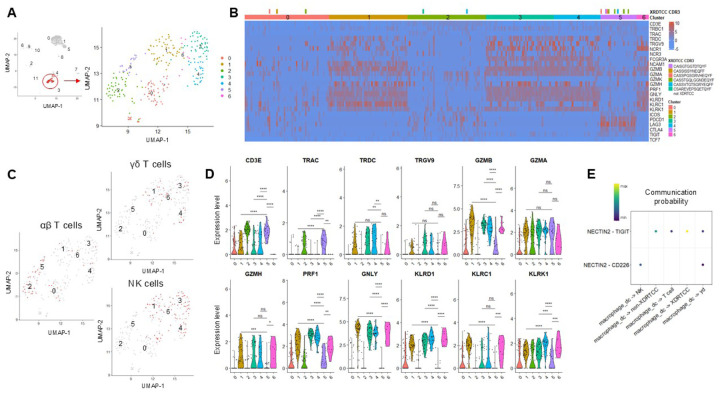
During rejection, γδ T cells and NK cells are found at high levels and express cytotoxic RNA profiles (**A**) Cluster 3 (see Fig.5A) was subset and re-clustered to examine additional heterogeneity, separating out into 6 clusters (**B**) XDRTCCs are found in Clusters 0, 2, and 5. XDRTCCs with identical clonotypes are annotated with identical color bars above the cluster identity metadata. Clusters containing XDRTCCs express some cytotoxic genes, though to a lesser extent than Clusters 1, 3, 4, and 6. These other clusters also express high levels of γδ T cell and NK cell-associated genes. (**C**) αβ T cells segregate out from γδ and NK cells which co-cluster. (**D**) Clusters 1, 3, 4, and 6 containing γδ and NK cells demonstrate higher levels of activation and cytotoxic genes than Clusters 0, 2, and 4 containing XDRTCCs. (**E**) Macrophages and DCs are predicted to interact with T cells by TIGIT, causing suppression, but NK and γδ T cells are predicted to interact via CD226, causing activation. *P < 0.05, ** P < 0.01, ***P < 0.001, ****P < 0.0001, by Wilcoxon Rank Sum test.

To dissect Cluster 3 at greater resolution, we re-clustered this cell subset (Fig.6A). The majority of XDRTCCs mapped onto Cluster 5 of this new UMAP (Fig.6B). Cluster 5 included some effector genes such as GZMA, GZMB and PRF1 as well as the costimulatory molecule ICOS and some coinhibitory molecules, including PDCD1, LAG3, CTLA4, and TIGIT (Fig.6B). Clusters 1, 3, 4, and 6 all demonstrated high expression of cytotoxic markers including GZMB, GZMA, GZMH, PRF1, and GNLY (Fig.6B). They also expressed high levels of TRDC and TRGV9, consistent with a γδ T cell phenotype (Fig.6B). Indeed, by transcriptional profiles, the innate and adaptive cells clustered separately , with αβ T cells (CD3E^+^ TRBC1^+^ TRDC^neg^) mainly in Clusters 0, 2, and 5 (Fig. 6C, Table S4), where XDRTCCs were located (Fig.6B). In contrast, γδ T cells (CD3E^+^ TRDC^+^) were detected mainly in Clusters 1, 3, and 4 and NK cells (CD3E^neg^ and FCGR3A^+^ or NCAM1^+^) were in Clusters 0, 1, 3, 4, and 6 (Fig.6C, Extended Data Fig8.B-C).

Given that γδ T cells also express unique TCRs, we examined their V gene usage. γδ T cells (CD3E^+^ TRDC^+^) identified in POD14L and in POD33K showed divergent TRGV gene usage (Extended Data Fig.9A-B), suggesting new recruitment of γδ T cells at POD33 rather than migration from POD14L. Indeed, γδ T cells at POD33 predominantly expressed TRGV9, consistent with peripheral blood origin (21). Furthermore, those in the POD33 kidney were phenotypically distinct, with elevated cytotoxic gene expression, including GZMB, GZMA, PRF1 and GNLY (Extended Data Fig.9C).

To compare the RNA profile of γδ and NK cells against αβ T cells, we first examined the expression of a T cell cytotoxicity pathway from MSigDB. Clusters containing γδ and NK cells tended to have higher expression of the signature than those containing αβ T cells (Extended Data Fig.9D). Clusters 1, 3, 4, and 6 containing γδ T cells and NK cells frequently expressed significantly higher levels of cytotoxic markers including GZMB, GZMH, PRF1, and GNLY than Cluster 5 containing the majority of αβ XDRTCCs (Fig.6D). Of note, GZMA levels were similar between clusters containing αβ as well as γδ and NK cells (Fig.6D).

We used the CellChat package algorithm to identify the likely cell-cell interactions of the different lymphocyte types. For the analysis, we used the same cell labelling strategy as in Extended Data Fig.5D, which was in agreement with SingleR predictions (Extended Data Fig.10A-B). CellChat predicted cell-cell interactions including the NECTIN2 pathway, which has two ligands mediating opposite effects on lymphocytes: TIGIT, which inhibits activation and CD226, which promotes cytotoxicity. Interestingly, macrophages and dendritic cells) were predicted to interact with XDRTCCs via TIGIT but with NK and γδ T cells via CD226, suggesting they activated cytotoxicity in innate lymphocytes (Fig.6E). Taken together, these data suggest that during an episode of acute rejection, innate lymphocytes as well as αβ T cells contribute significant cytotoxic effector function.

## Discussion

Here we report the first instance of xenogeneic donor-reactive T cell identification and tracking following a pig-to-human decedent organ transplant. We demonstrate marked and specific expansion of XDRTCCs in the circulation and detect them among leukocytes obtained from kidney biopsy specimens during AMR and from a lymphocyte collection prior to rejection, suggesting they may contribute to or even initiate AMR. We also obtained evidence for xenograft infiltration by activated recipient APCs and effector γδ T cells and NK cells. While recent xenotransplantation studies have highlighted the role of NK cell and macrophages in AMR(9, 10, 12, 22), ours is the first to characterize the adaptive donor-specific T cell repertoire in rejection, building upon our methods validated in human allograft recipients (23–25). Our study provides novel insights into human anti-pig responses *in vivo* and suggest that improved suppression of these pathways or tolerance induction may enhance the success of xenotransplantation.

The naïve human anti-pig xenoresponse, in contrast to the alloresponse, also includes strong indirect responses (3). In this study, we have established a method for characterizing the indirect response at the clonal level. We observe a potent primary indirect as well as direct xenoresponse, consistent with the notion that the extensive protein/peptide non-homology between pigs and humans causes strong and diverse immune reactions. Clonotypic analysis revealed minimal overlap between the sequences identified for the direct versus indirect CD4 or CD8 xenoresponse, indicating recognition of distinct specificities in each type of assay and arguing against semi-direct presentation as an explanation for the potent indirect response.

Clonotypic analyses of the decedent anti-donor pig response allowed us to identify XDRTCCs in the recipient and track them in the post-transplant circulation and xenograft. We detected selective expansion of CD4 and CD8 XDRTCCs following the transplant, most strikingly among CD8 XDRTCCs, among which a single, directly-xenoreactive clone represented > 20% of the repertoire by POD49. While this expansion of XDRTCCs occurred on a background of anti-thymocyte globulin-induced T cell lymphopenia which also led to the expansion of some non-XDRTCCs, the relative expansion of XDRTCCs was markedly greater (> 150-fold by POD49). These results demonstrate a marked *in vivo* T cell response to the source pig, a conclusion reinforced by the high level of clonal “convergence” for this subset, wherein increased numbers of unique clones encoding the same XDRTCC CDR3s were expanded after the transplant. The same directly reactive CD8 effector XDRTCCs found in the xenograft, in addition to directly and indirectly reactive CD4 clones, were detected as early as the POD14 lymphocele, suggesting that activation of XDRTCCs occurred in the lymphoid tissues prior to tissue infiltration. The detection of a Tfh XDRTCC in the perinephric lymphocele along with B cells raises the possibility that T-B interactions that preceded AMR took place in this collection in the absence of lymph nodes draining the transplanted kidney. Overall, the expansion of circulating XDRTCCs and their selective appearance in rejection biopsies suggest that the immunosuppression used was insufficient to fully suppress the anti-donor xenoresponse and raises the possibility that XDRTCCs were the initiators of innate immune infiltration and AMR in the xenograft.

We transplanted a composite pig thymus kidney graft based on our prior work demonstrating the ability of porcine thymic transplantation to tolerize murine T cells in immunocompetent mice (16, 17) and human T cells in a human immune system mouse model (14, 15). We have extended this approach to pig-to-non-human primate transplantation by generating composite thymokidney grafts in the prospective source pig (26, 27) or by transplanting vascularized porcine thymic lobes (28, 29). While recipient thymectomy was essential in murine models for tolerance mediated by porcine thymic xenografts (30, 31), the decedent transplant recipient was not thymectomized. Nevertheless, anti-donor MLR responses following the transplant gradually declined, with no change in anti-human responses (13). Thus, despite the expansion of XDRTCCs and their presence in the xenograft and the detection of minimal porcine thymic tissue at the time of experimental termination (13) it is possible that regulatory T cells derived from the thymus graft suppressed responses to the source pig, as noted in animal studies(15).

In addition to a role for T cells in kidney xenograft rejection, our studies also implicate innate NK cells and γδ T cells, both of which showed strong effector signatures in the graft during rejection. NK cells were prominent in subclinical humoral rejection in shorter-term pig-to-human decedent transplants (9), possibly activated due to the lack of human NK cell- inhibiting surface proteins in pigs (32). Studies of human anti-pig MLRs have demonstrated NK cell activation in the presence of IL-2 and/or whole PBMCs (33), presumably reflecting a requirement for cytokines produced by T cells. In our study, infiltrating XDRTCCs may have provided the cytokines needed for NK cell activation. We also observed a marked effector profile for γδ T cells in the rejection biopsy. The role of γδ T cells in xenotransplantation has been poorly characterized, though they were previously shown to play a critical role in rat to mouse bone marrow rejection (34). Of note, the immunosuppressive agents used in our study (Table S1) primarily suppress the adaptive rather than innate immune response. Further studies to understand the extent to which innate cellular components of the xenoresponse are dependent on T cell responses will determine the need only for improved T cell suppression versus a need to separately target innate immune response.

## Data Availability

Raw stimulated and unstimulated *TCRB* CDR3 data is available at Adaptive Biotechnologies’ immuneACCESS database. Raw single cell data is available on the NIH Sequence Read Archive (SAR) under SUB15009372.
